# When does working with AI empower or entrap university faculty? A dualistic passion perspective on job crafting

**DOI:** 10.3389/fpsyg.2026.1853560

**Published:** 2026-08-28

**Authors:** Jin Zhu, Yifei Zhang

**Affiliations:** School of Economics and Management, Jiangsu University of Science and Technology, Zhenjiang, China

**Keywords:** dualistic passion, human-AI collaboration, independent self-construal, job crafting, university faculty

## Abstract

**Introduction:**

Generative AI is now widely used in teaching, research, and academic writing, leading university faculty to engage in more sustained human-AI collaboration. However, existing research has largely focused on tool-related outcomes, offering limited insight into the psychological experiences and academic work practices associated with such collaboration.

**Methods:**

We conducted two survey-based studies using independent samples of Chinese university faculty. Study 1 (*n* = 252) examined whether human-AI collaboration engagement was associated with harmonious and obsessive passion, and whether independent self-construal moderated these associations. Study 2 (*n* = 363) replicated these relationships and further tested whether the two forms of passion were related to job crafting. Data were analyzed using partial least squares structural equation modeling.

**Results:**

Across both studies, human-AI collaboration engagement was positively associated with both harmonious and obsessive passion. Independent self-construal strengthened the association between collaboration engagement and harmonious passion, but not the association with obsessive passion. Study 2 further showed that harmonious passion was positively related to job crafting, whereas obsessive passion was negatively related to job crafting.

**Discussion:**

Human-AI collaboration may involve different psychological experiences among university faculty, depending on how such collaboration is internalized. Specifically, harmonious passion and greater job crafting reflect an empowerment-related pattern, whereas obsessive passion and reduced job crafting reflect a more constraining pattern of AI collaboration. These results point to the importance of motivational quality in explaining variation in academic work behavior beyond a tool-centered perspective.

## 1 Introduction

In recent years, the application of artificial intelligence (AI) in higher education has increasingly moved beyond instrumental support toward more sustained forms of cognitive collaboration. This shift has been accelerated by the development of generative AI and conversational systems, which are now used not only for information retrieval, process automation, and decision support, but also by many faculty members in core academic tasks such as knowledge generation, argument construction, teaching preparation, research development, and scholarly expression (Nguyen et al., 2024; Koroleva and Jogezai, 2025). Because academic work relies heavily on autonomous judgment, creative engagement, and academic identity, AI involvement may shape not only work processes but also how faculty members conduct and experience academic labor. In this context, many faculty members with generative AI experience are moving beyond occasional tool use; they increasingly evaluate AI outputs, engage in human-AI collaboration, and integrate AI into their teaching, research, and academic writing practices (Kang and Lou, 2022; Chiu and Rospigliosi, 2025).

However, scholarly understanding of AI in academic work has not fully kept pace with this shift. Existing research has largely followed a tool–outcome explanatory paradigm, focusing on whether AI use improves job satisfaction, trust, technology acceptance, or performance (Ma et al., 2024; Ali et al., 2025; Acosta-Enriquez et al., 2025; Heine and König, 2025). This perspective remains valuable, but it is less able to explain why faculty members who are similarly engaged in human-AI collaboration may experience it in very different ways. Some may experience AI collaboration as stimulating and self-enhancing, whereas others may experience it as difficult to disengage from or psychologically constraining. For university faculty, academic work is closely tied to professional judgment, self-expression, and the construction of academic identity (Djerasimovic and Villani, 2020; Othman and Lo, 2023). Therefore, AI collaboration is not simply an external tool-use behavior; it may become internalized as part of how academics understand, regulate, and enact their professional selves. Accordingly, the key question is not only whether faculty engage with AI, but how such engagement is motivationally internalized.

Against this background, the dualistic model of passion provides a particularly appropriate theoretical lens for examining how human-AI collaboration is motivationally internalized by university faculty members. Prior perspectives on motivation and work design, such as self-determination theory (Ryan et al., 2022) and the job demands–resources model (Bakker et al., 2023), offer important insights into autonomous motivation and the resource–demand nature of work experiences. However, this study is concerned less with motivation in general or with whether AI functions as a work resource or demand, and more with how sustained collaboration with AI becomes incorporated into university faculty members’ sense of self as a meaningful work activity. The dualistic model of passion is therefore well suited to this question, because it distinguishes between harmonious passion and obsessive passion as two fundamentally different paths of motivational internalization. It emphasizes that passion is not merely a reflection of engagement intensity, but depends on whether individuals internalize an activity into the self in an autonomous or controlled manner (Vallerand et al., 2003). Harmonious passion enables individuals to engage flexibly in their work while maintaining psychological balance, whereas obsessive passion is characterized by high involvement accompanied by persistent internal pressure and rigid persistence (Curran et al., 2015). By applying this model to human-AI collaboration, this study can explain why similar levels of collaboration engagement may be associated with both academic empowerment and psychological constraint.

Furthermore, the experience of different types of passion in human-AI collaboration may not depend solely on collaboration intensity, but also on how faculty members define the relationship between the self and academic work. Although university faculty generally enjoy a relatively high level of professional autonomy, prior research has shown that they differ in the extent to which autonomy, self-direction, and personal goal attainment serve as core foundations of their work motivation (Bentley and Kyvik, 2013; Niemczyk and Rónay, 2023). Independent self-construal captures these differences by reflecting the extent to which individuals define themselves in terms of autonomy, uniqueness, self-expression, and personal agency (Singelis, 1994; Kitayama et al., 1997; Voyer and Franks, 2014). Because the psychological meaning of AI collaboration may be shaped by users’ cultural and social orientations (Lu et al., 2025; Sugitani et al., 2025), faculty members with stronger independent self-construal may be more likely to interpret AI collaboration as a self-chosen resource that supports their academic goals, rather than as a merely external technological demand. Therefore, independent self-construal may serve as an important boundary condition in the association between human-AI collaboration engagement and different forms of work passion.

However, identifying different types of passion alone remains insufficient to reveal the academic consequences of human-AI collaboration. Because harmonious and obsessive passion reflect different ways in which university teachers internalize AI-related academic activities, these motivational experiences may also be associated with how teachers organize, adjust, and give meaning to their academic work. Job crafting provides a relevant behavioral lens for understanding such work-related adjustments, as it refers to teachers’ self-initiated modifications of the task, relational, and cognitive boundaries of their work, including what they do, how they interact with others, and how they interpret work meaning (Wrzesniewski and Dutton, 2001). In higher education contexts, job crafting is not an occasional proactive behavior undertaken by individuals; rather, it constitutes a routine practice mechanism embedded in highly autonomous and relatively loosely structured academic work. Through this mechanism, university teachers continuously adjust their tasks, relationships, and constructions of work meaning (McNaughtan et al., 2022). As a relatively stable motivational orientation, passion may be associated with how teachers modify their work content, reconstruct academic relationships, and delineate work boundaries (Vallerand, 2016; Kibaroğlu et al., 2025). Therefore, the proposed association between passion and job crafting constitutes a basis for understanding how human-AI collaboration may be linked to university teachers’ academic practice.

**Figure 1:**
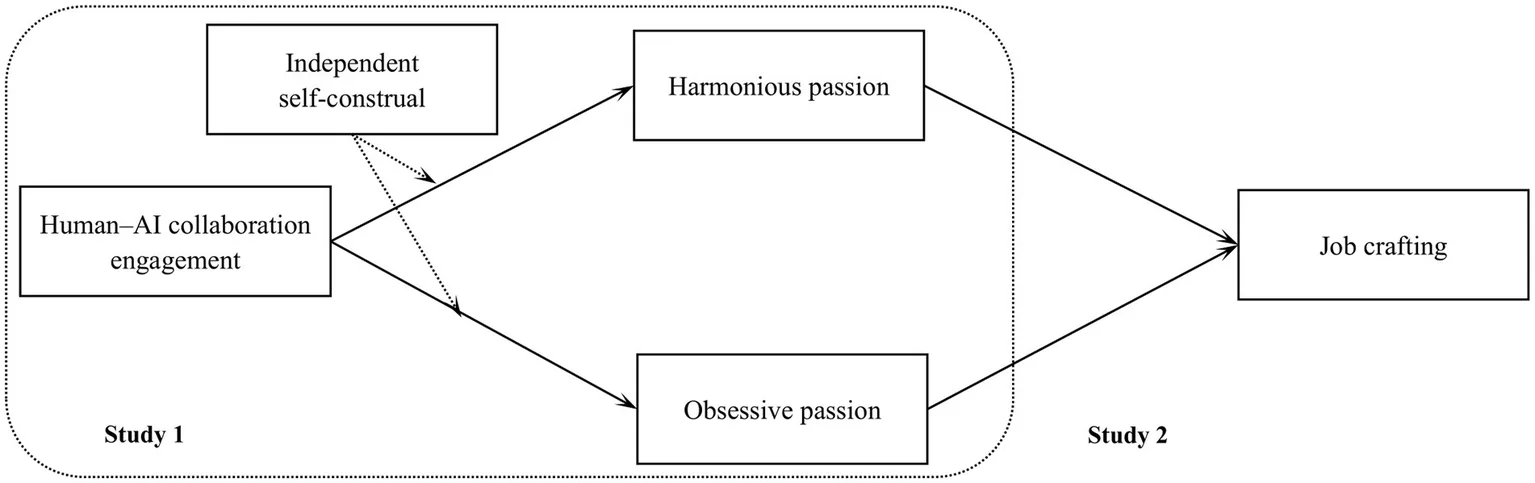
The conceptual model of this study.

Building on this framework, we conducted two survey-based studies among university faculty with experience using generative AI. Study 1 examined whether human-AI collaboration engagement was associated with harmonious and obsessive passion, and whether independent self-construal moderated these relationships. Study 2 replicated these relationships in an independent sample and further tested whether the two forms of passion were differentially associated with job crafting. Through this design, this research seeks to advance the study of human-AI collaboration beyond a tool–outcome perspective by highlighting motivational internalization and its behavioral implications. The conceptual models for Study 1 and Study 2 are shown in Figure 1.

## 2 Study 1: human-AI collaboration and dualistic passion

### 2.1 Theoretical background and hypotheses development

Study 1 examines university teachers’ psychological responses to human-AI collaboration and develops the corresponding hypotheses. To this end, we integrate the dualistic model of passion with self-construal to explain why teachers may develop distinct types of passion experiences when engaging in deep collaboration with AI. In contrast to prior research that has primarily focused on the outcomes of AI use, our analysis centers on the processual experience of participation in human-AI collaboration. Specifically, we examine how such engagement is associated with university teachers’ work passion in ways that are theoretically consistent with different forms of motivational internalization.

The dualistic model of passion posits that when individuals internalize work activities into the self in an autonomous manner, they are more likely to develop harmonious passion, which is characterized by flexible engagement, positive experiences, and a healthy psychological balance (Vallerand et al., 2003; Curran et al., 2015). Empirical studies in knowledge-intensive and creative professions have shown that when technologies or job resources are perceived as supportive tools for achieving personal goals, they are positively associated with harmonious passion (Ding et al., 2023; Lin et al., 2024; Yang et al., 2026). These findings suggest that the critical factor lies not in the intensity of engagement perse, but in whether such engagement stems from autonomous internalization. In the context of university teaching, participation in human-AI collaboration may enhance individuals’ sense of control and autonomy over their academic work by providing cognitive support, alleviating repetitive burdens, and expanding opportunities for scholarly exploration (Sowa et al., 2021; Renkema and Tursunbayeva, 2024; Zheng et al., 2025). When teachers perceive AI as a collaborative resource that serves their academic goals and research interests, collaboration with AI is more likely to be experienced as autonomously meaningful and may therefore be positively associated with harmonious passion (Guo et al., 2025).

The dualistic model of passion further suggests that when work activities are internalized into the self in a controlled manner, individuals, even when highly engaged, are more likely to develop obsessive passion, which is characterized by internal pressure, rigid persistence, and difficulty achieving psychological detachment (Vallerand et al., 2003; Curran et al., 2015). Some research conducted in performance-oriented and evaluation-intensive work environments has shown that high levels of engagement do not necessarily result in positive psychological outcomes (Liao et al., 2025; Hai et al., 2025; Chuang et al., 2025). Instead, when work demands become closely intertwined with one’s self-worth and individuals experience a persistent sense of internal obligation, obsessive passion is more likely to emerge, often accompanied by emotional exhaustion and work–life conflict. In the context of human-AI collaboration, the constant availability and high responsiveness of AI may intensify individuals’ self-monitoring and internal performance expectations (Hai et al., 2025; Chuang et al., 2025). For university teachers, when collaboration with AI is accompanied by psychological anxiety, adaptation pressure, and sustained high levels of technological involvement, it may be experienced as a resource-depleting job demand rather than a manageable resource. This dynamic is particularly pronounced in the absence of sufficient organizational support, where teachers must continually invest time and effort to fulfill the implicit obligations associated with AI use in teaching and research (Tan et al., 2025; Hu et al., 2025). Under such conditions, participation in human-AI collaboration may be associated with controlled forms of motivational experience and obsessive passion. Thus, we propose the following hypotheses:

*H1a*: Human-AI collaboration engagement is positively related to harmonious passion

*H1b*: Human-AI collaboration engagement is positively related to obsessive passion

Although participation in human-AI collaboration creates the conditions for the development of different types of passion, its psychological consequences do not occur uniformly across individuals. How individuals define the relationship between the self and their work systematically shapes the manner in which motivation is internalized (Kitayama et al., 1997; Voyer and Franks, 2014). In particular, independent self-construal emphasizes autonomy, self-direction, and the pursuit of personal goals, characteristics that are especially salient in academic professions (Singelis, 1994). In work contexts, self-construal may interact with task and contextual characteristics to shape employees’ motivational and behavioral responses. For example, independent self-construal has been associated with stronger career commitment and career-oriented proactive behavior under conditions of high job autonomy, whereas change-related stress may become more salient when organizational changes affect aspects of work that are central to the personal self (Wisse and Sleebos, 2016; Wu et al., 2018; Zhang and Li, 2024).

On the one hand, teachers with a high level of independent self-construal are more likely to perceive human-AI collaboration as a self-chosen resource that expands their personal capabilities and facilitates the achievement of academic goals, thereby reinforcing autonomous internalization and enhancing harmonious passion (Vallerand et al., 2003; Curran et al., 2015; Sowa et al., 2021; Renkema and Tursunbayeva, 2024). On the other hand, independent self-construal may also shape how teachers respond to the more demanding side of human-AI collaboration. Because obsessive passion reflects controlled internalization accompanied by internal pressure, rigid persistence, and difficulty disengaging from an activity, AI collaboration may be more likely to take on a controlled motivational meaning when it is embedded in pressure-laden academic contexts, such as continuous technological adaptation, institutional expectations for AI integration, and performance norms surrounding AI-supported teaching and research (Vallerand et al., 2003; Curran et al., 2015; Chuang et al., 2025; Tan et al., 2025; Hu et al., 2025). Accordingly, we propose the following moderating hypotheses:

*H2a*: Independent self-construal moderates the relationship between human-AI collaboration engagement and harmonious passion.

*H2b*: Independent self-construal moderates the relationship between human-AI collaboration engagement and obsessive passion.

### 2.2 Methodology

#### 2.2.1 Sample and procedure

This study employed a purposive sampling strategy to recruit in-service faculty members from universities in mainland China who had experience using generative AI in academic work. Participants were primarily drawn from comprehensive universities and undergraduate institutions, where teaching, research, and academic writing constitute core faculty responsibilities. At the beginning of the questionnaire, respondents were screened for prior generative AI use. To be eligible, they had to have used generative AI tools in at least one academic work context, including teaching, research, academic writing, or other academic tasks. Respondents who reported no prior experience with generative AI were screened out. No additional minimum frequency threshold was imposed beyond prior use experience.

The survey link was distributed through academic communities, faculty networks, and scholarly communication platforms to reach faculty members with relevant academic backgrounds and AI-use experience. To reduce potential common method bias (CMB), participants were assured of anonymity and informed that there were no right or wrong answers. Participation was voluntary, and no monetary compensation was provided. The data collection was conducted over one month, starting in October 2025. A total of 305 responses were collected. Responses were excluded if they were incomplete, failed the directed-response attention-check item, showed excessively short completion times, or contained patterned or inconsistent answers. The attention-check item asked respondents to select a designated response option to indicate that they were paying attention. After applying these screening rules, 252 valid responses were retained for subsequent analysis.

The final sample had a relatively balanced gender distribution, including female participants (50.8%) and male participants (49.2%). Regarding age, participants were distributed across four groups: under 30 years old (35.7%), 30–39 years old (27.8%), 40–49 years old (22.6%), and 50 years old or above (13.9%). In terms of academic position, the sample included lecturers/assistant professors (37.7%), associate professors (36.5%), full professors (17.1%), and participants holding other academic positions (8.7%). Regarding teaching experience, participants had less than 5 years of experience (42.1%), 5–10 years of experience (22.2%), 11–20 years of experience (23.8%), or more than 20 years of experience (11.9%). The sample covered multiple academic disciplines, including humanities (29.0%), social sciences (25.4%), natural sciences (15.1%), engineering/technology (16.3%), medicine/health sciences (9.9%), and other fields (4.4%). Regarding AI-use frequency, participants reported using generative AI only for occasional experimentation (10.7%), occasionally for specific tasks (15.1%), regularly as part of their work (32.5%), or very frequently across many tasks (41.7%).

#### 2.2.2 Measures

Except for demographic variables, all core constructs were measured using established scales. These instruments were contextually adapted to reflect university faculty members’ academic work settings and the characteristics of generative AI use. All items were rated on a seven-point Likert scale ranging from 1 = strongly disagree to 7 = strongly agree. The adaptation process followed three steps. First, the original English items were translated into Chinese and then checked against the original wording to ensure semantic equivalence. Second, the wording was reviewed by the research team to assess whether the adapted items were appropriate for university faculty members’ teaching, research, academic writing, and AI-use contexts. Third, the adapted items were examined for clarity and contextual relevance before the formal survey administration. The final adapted items are reported in the Supplementary Material. The specific measures are described below.

Human-AI collaboration engagement was measured by adapting the engagement-related items from the CI-Tex Scale developed by Zabel et al. (2025). In the original scale, these items capture users’ active engagement behaviors when collaborating with text-based conversational agents, such as rephrasing requests, seeking infozrmation, asking for help, brainstorming, learning, managing tasks, paying for use, adjusting workflows, and consulting agents before important decisions. In this study, these items were adapted to the context of university faculty members’ academic work. Specifically, references to conversational agents were replaced with “AI” or “AI tools”, and the usage context was restricted to teaching, research, academic writing, and other academic tasks. The adapted scale consists of 11 items. Although the items cover different behavioral expressions, they were retained as indicators of human-AI collaboration engagement because they correspond to the engagement dimension of the original CI-Tex Scale and reflect varying degrees of active involvement in AI-supported academic work.

Harmonious passion was measured using the harmonious passion subscale of the Passion Scale developed by Vallerand et al. (2003). The original instrument assesses individuals’ autonomous motivation and harmonious engagement in a given activity. In this study, the target activity was specified as “using AI in academic work” to capture faculty members’ harmonious passion in the context of AI use. The adapted scale consists of 7 items reflecting positive emotions, a sense of self-congruence, and the perceived compatibility of AI use with other work and life activities.

Obsessive passion was measured using the obsessive passion subscale of the same Passion Scale developed by Vallerand et al. (2003). Following the same contextual adaptation procedure, the items were revised to focus on faculty members’ experiences when using AI in academic work, particularly feelings of loss of control, emotional dependence, and compulsive tendencies. The revised subscale includes 7 items assessing emotional reliance on AI and difficulties in regulating the urge to use it.

Independent self-construal was measured using the independent self-construal subscale of the self-construal scale developed by Singelis (1994), which consists of 12 items. This scale assesses individuals’ tendencies regarding self-identity, autonomy, and self-expression. The original items were retained without contextual modification to preserve their function as a stable individual trait measure.

Consistent with prior research, several demographic and behavioral variables that may influence the relationships among the focal constructs were controlled for in the analyses, including age, gender, and AI usage frequency (Cheng et al., 2023; Kong et al., 2024; Brynjolfsson et al., 2025). These variables were entered as control variables in subsequent statistical models to enhance the robustness of the findings.

In addition, to assess potential CMB at the statistical analysis stage, a marker variable was introduced. Specifically, a five-item scale capturing general life preference tendencies was used as the marker variable. This construct reflects individuals’ overall preference for routine and predictability in daily life and is conceptually unrelated to human-AI collaboration, work passion, self-construal, or job crafting. Such a marker variable shares a similar response process with the substantive measures while remaining conceptually distinct, thereby serving as an appropriate proxy for method variance (Simmering et al., 2015; Leonard et al., 2024).

#### 2.2.3 Data analysis strategy

Given the analytical purpose of this study, PLS-SEM was used to estimate the proposed latent-variable model. Although the research model was theoretically grounded and relatively parsimonious, the study examined human-AI collaboration in an emerging academic work context, and several established scales were contextually adapted to capture university faculty members’ experiences with generative AI. Accordingly, the analysis focused not only on testing theoretically derived associations but also on assessing the explanatory and predictive relevance of the model for endogenous constructs. PLS-SEM was considered appropriate for this purpose because it emphasizes variance explanation, accommodates latent constructs measured by multiple indicators, and provides bootstrapped estimates for direct and moderating relationships (Hair et al., 2019; Sarstedt et al., 2022).

All constructs were specified as reflective measurement models, as the observed items were treated as manifestations of their corresponding latent constructs rather than as defining components of the constructs. The analysis was conducted using SmartPLS 4.1. The measurement model was first assessed by examining indicator loadings, Cronbach’s *α*, composite reliability, average variance extracted, the Fornell-Larcker criterion, and HTMT values. The structural model was then evaluated using path coefficients, 5,000 bootstrap resamples, confidence intervals, *p* values, *r^2^*, *q^2^*, and VIF values. The moderating effect of independent self-construal was tested by adding an interaction term between human-AI collaboration engagement and independent self-construal. Age, gender, and AI usage frequency were included as control variables.

### 2.3 Results

#### 2.3.1 CMB evaluation

Given the single-source and cross-sectional design of Study 1, we examined CMB using three complementary approaches. Harman’s single-factor test showed that the first unrotated factor explained 27.3% of the total variance, below the 50% threshold. The common method factor test further showed that only a few method-factor loadings were significant and generally small, with the substantive constructs explaining much more variance than the method factor on average, 0.662 versus 0.024. In addition, the marker variable analysis indicated that the paths from the marker variable to the key endogenous variables were not significant. After controlling for the marker variable, the adjusted *r^2^* values changed only slightly, and the main structural paths remained stable in direction, significance, and magnitude. Taken together, these results suggest that serious CMB was not evident in Study 1 (Podsakoff et al., 2003; Liang et al., 2007; Rönkkö and Ylitalo, 2011).

#### 2.3.2 Measurement model analysis

All standardized factor loadings of the measurement items met the recommended criteria, ranging from 0.724 to 0.899 and exceeding the threshold of 0.70. This indicates strong associations between the items and their respective latent constructs. At the construct level, all latent variables demonstrated satisfactory internal consistency reliability, with Cronbach’s alpha and composite reliability (CR) values exceeding the recommended threshold of 0.70. Regarding convergent validity, the average variance extracted (AVE) for each construct was above 0.50, ranging from 0.587 to 0.700, indicating that the latent constructs explained the majority of the variance in their indicators (Hair et al., 2019). Discriminant validity results are presented in Table 1. The square root of each construct’s AVE was greater than its correlations with other constructs. In addition, all heterotrait–monotrait ratio (HTMT) values were below the conservative threshold of 0.85, further supporting adequate discriminant validity among the constructs. Therefore, the measurement model met established evaluation criteria and provided a reliable foundation for subsequent structural model analysis (Henseler et al., 2015).

**Table 1:** Descriptive statistics and discriminant validity analysis.

Constructs	Mean	S.D.	Skewness	Kurtosis	Fornell-Larcker criterion				HTMT ratio			
					1	2	3	4	1	2	3	4
1. HCE	3.942	1.290	0.009	−1.196	**0.766**				/			
2. HP	3.702	1.442	0.171	−1.296	0.537	**0.837**			0.563			
3. IS	3.877	1.512	−0.186	−1.605	−0.023	0.108	**0.823**		0.069	0.088		
4. OP	3.982	1.422	−0.006	−1.284	0.465	0.217	−0.000	**0.825**	0.488	0.230	0.052	/

The bold elements are the square roots of AVE, HCE, Human-AI collaboration engagement; HP, Harmonious passion; IS, Independent self-construal; OP, Obsessive passion.

#### 2.3.3 Structural model analysis and hypothesis testing

This study employed SmartPLS 4.1 to analyze the structural model and used PLS-SEM to test the research hypotheses. The model’s explanatory power and predictive relevance were assessed using the coefficient of determination (*r^2^*) and the Stone–Geisser *q^2^* statistic, respectively. The results indicated that the *r^2^* values for the endogenous constructs ranged from 0.227 to 0.389, while all *q^2^* values were greater than zero, suggesting acceptable explanatory power and satisfactory predictive relevance of the model. In addition, the variance inflation factor (VIF) values for all paths were below 5 and well under the recommended threshold, indicating the absence of multicollinearity concerns (Hair et al., 2019).

The significance of the path coefficients was evaluated using a bootstrapping procedure with 5,000 resamples. Table 2 presents the hypothesis testing results after controlling for gender, age, and AI usage frequency. The results show that human-AI collaboration engagement was positively associated with harmonious passion (*β* = 0.540, *p* < 0.001) and obsessive passion (*β* = 0.464, *p* < 0.001), supporting H1a and H1b.

**Table 2:** Results of hypothesis testing.

Paths	*β*-value	*LLCI*	*ULCI*	*p*-value	*r^2^*	*q^2^*	VIF	Support
Panel A. Results of the direct effect.								
H1a: HCE → HP	0.540	0.449	0.634	<0.001	0.298	0.195	1.003	Yes
H1b: HCE → OP	0.464	0.359	0.574	<0.001	0.227	0.137	1.003	Yes
Panel B. Results of the moderating effect.								
H2a: IS × HCE → HP	0.275	0.103	0.382	<0.001	0.389	0.258	1.018	Yes
H2b: IS × HCE → OP	0.017	−0.107	0.127	0.779	0.227	0.134	1.018	No

HCE, Human-AI collaboration engagement; HP, Harmonious passion; IS, Independent self-construal; OP, Obsessive passion; LLCI, Lower-limit of confidence interval; ULCI, Upper-limit of confidence interval.

**Figure 2:**
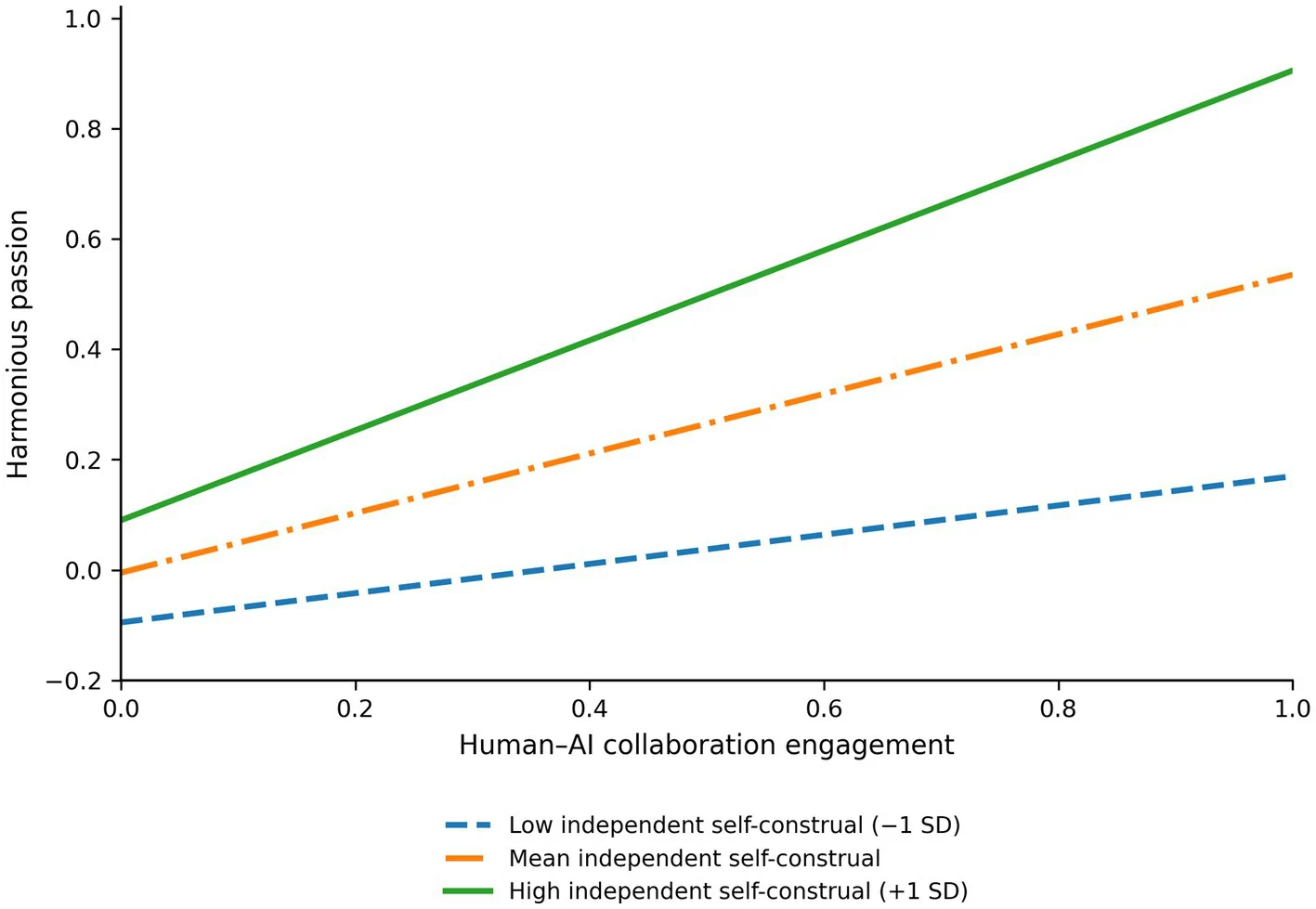
Moderating effect of independent self-construal on the relationship between human–AI collaboration engagement and harmonious passion.

The moderating effects of independent self-construal were subsequently examined. The interaction between independent self-construal and human–AI collaboration engagement was positively associated with harmonious passion (*β* = 0.275, *p* < 0.001), supporting H2a. By contrast, the interaction term was not significantly associated with obsessive passion (*β* = 0.017, *p* = 0.779); thus, H2b was not supported. To further interpret the significant moderating effect, a simple-slope analysis was conducted. As illustrated in Figure 2, the positive association between human–AI collaboration engagement and harmonious passion was weaker at a low level of independent self-construal (−1 *SD*), stronger at the mean level, and strongest at a high level of independent self-construal (+1 *SD*). The progressively steeper slopes indicate that independent self-construal strengthened the positive association between human–AI collaboration engagement and harmonious passion.

### 2.4 Discussion

Study 1 shows that faculty engagement with AI is associated with two distinct forms of work passion rather than a single psychological experience. Human-AI collaboration engagement was positively associated with both harmonious and obsessive passion, indicating that the same collaborative activity may be internalized in different motivational ways. The results lend support to the dualistic model of passion, which posits that a single activity may be internalized in either an autonomous or a controlled manner (Vallerand et al., 2003; Curran et al., 2015). Furthermore, they indicate that in work contexts heavily reliant on professional judgment and academic identity, human-AI collaboration inherently carries psychological duality. In other words, a high level of engagement in human-AI collaboration does not inherently imply either positive or negative psychological outcomes; rather, its psychological meaning depends on how the collaborative experience is integrated into the individual’s self-system.

Further moderation analyses demonstrated that independent self-construal plays a selective role in these associations. Specifically, independent self-construal significantly strengthened the positive relationship between human-AI collaboration engagement and harmonious passion, but did not significantly moderate the association between collaboration engagement and obsessive passion. This asymmetric pattern indicates that independent self-construal mainly helps explain the autonomous internalization pathway, whereby faculty members interpret human-AI collaboration as a self-chosen academic resource and develop stronger harmonious passion. By contrast, the non-significant moderation for obsessive passion suggests that controlled internalization may be less attributable to self-construal alone and more closely related to pressure-laden academic conditions, including technological adaptation, institutional expectations for AI integration, and performance norms surrounding AI-supported teaching and research. These findings suggest that the psychological consequences of human-AI collaboration depend not only on collaboration intensity, but also on the quality of motivational internalization.

## 3 Study 2: dualistic passion and job crafting

### 3.1 Theoretical extension and hypotheses development

In the preceding analysis, work passion was conceptualized as the manner in which university faculty internalize academic activities as part of the self, rather than merely as a level of engagement (Vallerand et al., 2003; Curran et al., 2015). Different types of passion imply that faculty members organize their relationship with academic work in distinct ways: either as an activity that can be autonomously regulated and temporarily disengaged from, or as an obligation characterized by persistent self-imposed demands and difficulty in detachment. Importantly, such internalization does not remain confined to the psychological level; it may also be reflected in how faculty conduct their daily academic work. Thus, the significance of work passion lies not in its mere presence but in how it is associated with the individual’s actual patterns of interaction with work.

In the context of university faculty, shifts in interaction patterns are typically not reflected in the quantity of tasks completed, but rather in ongoing adjustments to the structure, boundaries, and meaning of work. Academic roles commonly encompass multiple responsibilities—including research, teaching, and academic service—which are often loosely structured organizationally, requiring faculty members to autonomously coordinate their priorities and pacing (Culver et al., 2026; Yang and Si, 2025). Within this process, job crafting represents a highly characteristic behavioral response among university faculty. By proactively reshaping task boundaries, academic relationships, and the construction of work meaning, individuals are able to regulate their relationship with academic work within existing institutional and resource constraints (McNaughtan et al., 2022; Xu et al., 2024). Accordingly, job crafting provides a behavioral lens that is closely aligned with the motivational internalization logic discussed earlier, offering a concrete framework for understanding how different types of work passion are enacted in academic practice.

Different types of work passion are expected to be associated with job crafting in distinct ways. Harmonious passion reflects a relatively autonomous internalization of academic activities into the self, enabling individuals to remain highly engaged in their work while still retaining the capacity to regulate work pace and boundaries (Vallerand, 2016; Zhang et al., 2025; Kibaroğlu et al., 2025). Under such conditions, faculty members are more likely to view job crafting as an important means of maintaining alignment between work and the self. Through proactively adjusting task arrangements, collaboration patterns, or the construction of work meaning, they can preserve flexibility and sustainability in their academic practice. Existing research indicates that harmonious passion is closely associated with exploratory behaviors, self-regulation, and adaptive behavioral adjustments—characteristics that closely align with the behavioral essence of job crafting (Zhang et al., 2025; Liao et al., 2025; Kibaroğlu et al., 2025). Accordingly, when university faculty engage in their academic work with harmonious passion, they are more likely to actively reshape their work approaches through job crafting behaviors.

In contrast, obsessive passion reflects a controlled form of internalization, under which individuals tend to experience academic activities as ongoing self-imposed demands rather than as work practices that can be flexibly regulated (Vallerand, 2016; Kenyhercz et al., 2026). Although obsessive passion is likewise accompanied by high levels of involvement, its behavioral consequences are typically manifested in the reinforcement of existing work arrangements rather than in the restructuring of work itself (Gülbahar and Özkan, 2023; Kenyhercz et al., 2026). This behavioral pattern does not imply a lack of action; rather, it reflects rigidity in action—individuals are more inclined to maintain established tasks and role boundaries, thereby limiting opportunities for proactive adjustments to their work approaches. Prior research suggests that obsessive passion does not reliably promote adaptive job crafting behaviors, and its relationship with job crafting may be negative or substantially weaker than that of harmonious passion (Kenyhercz et al., 2026; Zhang et al., 2025). Accordingly, this study proposes the following hypotheses:

*H3a*: Harmonious passion is positively related to job crafting

*H3b*: Obsessive passion is negatively related to job crafting

### 3.2 Methodology

#### 3.2.1 Sample and procedure

Study 2 was conducted using an independent sample distinct from that in Study 1. Rather than merely replicating prior direct relationships, this study aims to examine an extended pattern of associations among human-AI collaboration engagement, different forms of work passion, and behavioral outcomes. Collecting data from a separate sample within the same target population strengthens the robustness of the findings and reduces potential concerns regarding sample overlap. Consistent with Study 1, this study focused on in-service faculty members from universities in mainland China who had experience using generative AI in academic work. The same eligibility criteria, AI-use screening rule, response-quality screening procedures, attention-check procedure, and compensation policy used in Study 1 were applied.

Purposive sampling was employed, and the questionnaire was distributed through academic communities, departmental communication networks, and peer referrals to reach faculty members with relevant academic backgrounds and AI-use experience. Participants were primarily recruited from comprehensive universities and undergraduate institutions, where teaching, research, and academic writing are central faculty responsibilities. The data collection was conducted over approximately two months, from December 2025 to January 2026. A total of 427 responses were obtained. After excluding incomplete questionnaires, failed attention checks, unusually short completion times, and other invalid responses, 363 valid cases were retained for analysis.

The final sample had a relatively balanced gender distribution, including female participants (49.6%) and male participants (50.4%). Regarding age, participants were distributed across four groups: under 30 years old (28.4%), 30–39 years old (35.3%), 40–49 years old (24.5%), and 50 years old or above (11.8%). In terms of academic position, the sample included lecturers/assistant professors (32.2%), associate professors (46.3%), full professors (14.0%), and participants holding other academic positions (7.4%). Regarding teaching experience, participants had less than 5 years of experience (32.2%), 5–10 years of experience (32.2%), 11–20 years of experience (26.2%), or more than 20 years of experience (9.4%). The sample covered multiple academic disciplines, including humanities (24.5%), social sciences (22.9%), natural sciences (19.0%), engineering/technology (19.8%), medicine/health sciences (8.8%), and other fields (5.0%). Regarding AI-use frequency, participants reported using generative AI only for occasional experimentation (8.8%), occasionally for specific tasks (17.1%), regularly as part of their work (35.8%), or very frequently across many tasks (38.3%).

#### 3.2.2 Measures

Except for job crafting, all measures used in Study 2 were consistent with those in Study 1, including human-AI collaboration engagement, harmonious passion, obsessive passion, independent self-construal, demographic variables, AI-use frequency, and the marker variable. This ensured comparability between the two studies while allowing Study 2 to extend the model by introducing job crafting as an additional behavioral outcome. The same response format and adaptation principles described in Study 1 were applied.

Job crafting was measured using the scale developed by Leana et al. (2009). Although the original scale was designed primarily for classroom and early education contexts, the items were adapted to reflect university faculty members’ teaching, research, and broader academic work activities while preserving the core meaning of proactive adjustment of one’s work approach.

#### 3.2.3 Data analysis strategy

Study 2 employed PLS-SEM to test the extended research model, following the same analytical procedure and modeling decisions described in Study 1. The analysis first assessed potential CMB, followed by an evaluation of the measurement model’s reliability and validity. On this basis, the structural path relationships between harmonious passion, obsessive passion, and job crafting behavior were examined. Control variables were incorporated into the analysis, and the marker variable technique was applied to further assess the potential influence of CMB.

### 3.3 Results

#### 3.3.1 CMB evaluation

Given that Study 2 also relied on single-source survey data, we replicated the procedures used in Study 1 to assess CMB. Harman’s single-factor test showed that the first unrotated factor explained 22.9% of the total variance, below the 50% threshold. The common method factor test further indicated that only a limited number of method-factor loadings were significant, and their coefficients were generally small. In terms of explained variance, the substantive constructs explained substantially more variance on average than the method factor, 0.666 versus 0.016, suggesting that method effects accounted for only a minor proportion of the overall variance (Liang et al., 2007). In addition, the marker variable analysis showed that the paths from the marker variable to the key endogenous variables were not significant. After controlling for the marker variable, the direction, significance, and effect sizes of the main structural paths remained stable, and the model’s explanatory power showed no substantive change. Thus, these results suggest that serious CMB was not evident in Study 2 (Podsakoff et al., 2003; Liang et al., 2007; Rönkkö and Ylitalo, 2011).

#### 3.3.2 Measurement model analysis

At the indicator level, all standardized factor loadings exceeded the recommended threshold. At the construct level, Cronbach’s *α* and CR values were all above 0.70, and the AVE values exceeded 0.50, indicating satisfactory internal consistency reliability and convergent validity (Hair et al., 2019). Regarding discriminant validity, as shown in Table 3, the square roots of the AVE values for each construct were greater than their correlations with other constructs. In addition, all HTMT values were below the recommended threshold, further supporting adequate discriminant validity among the constructs (Henseler et al., 2015).

**Table 3:** Descriptive statistics and discriminant validity analysis.

Constructs	Mean	S.D.	Skewness	Kurtosis	Fornell-Larcker criterion					HTMT ratio				
					1	2	3	4	5	1	2	3	4	5
1. HCE	3.949	1.271	−0.031	−1.156	**0.762**					/				
2. HP	3.890	1.434	−0.033	−1.328	0.561	**0.831**				0.594				
3. IS	3.736	1.587	0.021	−1.568	−0.057	0.034	**0.844**			0.073	0.042			
4. JC	3.584	1.337	0.272	−0.965	0.207	0.413	0.101	**0.818**		0.215	0.449	0.094		
5. OP	3.936	1.373	0.047	−1.200	0.518	0.336	−0.044	0.015	**0.820**	0.549	0.362	0.049	0.049	/

The bold elements are the square roots of AVE, HCE, Human-AI collaboration engagement; HP, Harmonious passion; IS, Independent self-construal; OP, Obsessive passion; JC, Job crafting.

#### 3.3.3 Structural model analysis and hypothesis testing

This study likewise employed SmartPLS 4.1 and applied PLS-SEM to analyze the proposed model. First, the *r^2^* values of the endogenous variables ranged from 0.191 to 0.393, while all *q^2^* values exceeded zero, indicating acceptable explanatory power and satisfactory predictive relevance of the model. In addition, all VIF values were below 5, suggesting that multicollinearity was not a concern (Hair et al., 2019).

The significance of the path coefficients was assessed using bootstrapping with 5,000 resamples. Table 4 presents the results of the hypothesis testing after controlling for gender, age, and AI usage frequency. The findings indicate that human-AI collaboration engagement was positively associated with harmonious passion (*β* = 0.561, *p* < 0.001) and obsessive passion (*β* = 0.518, *p* < 0.001), supporting H1a and H1b. Further analyses reveal that harmonious passion is positively related to job crafting (*β* = 0.462, *p* < 0.001), whereas obsessive passion is negatively associated with job crafting (*β* = −0.138, *p* < 0.01), providing support for H3a and H3b.

**Table 4:** Results of hypothesis testing.

Paths	*β*-value	*LLCI*	*ULCI*	*p*-value	*r^2^*	*q^2^*	VIF	Support
Panel A. Results of the direct effect.								
H1a: HCE → HP	0.561	0.486	0.636	<0.001	0.315	0.231	1.000	Yes
H1b: HCE → OP	0.518	0.434	0.604	<0.001	0.268	0.176	1.000	Yes
H3a: HP → JC	0.462	0.378	0.553	<0.001			1.141	Yes
H3b: OP → JC	−0.138	−0.230	−0.047	0.004	0.191	0.122	1.144	Yes
Panel B. Results of the moderating effect.								
H2a: IS × HCE → HP	0.286	0.117	0.359	<0.001	0.393	0.267	1.008	Yes
H2b: IS × HCE → OP	0.005	−0.095	0.100	0.917	0.269	0.176	1.008	No
Panel C. Specific indirect effects.								
HCE → OP → JC	−0.072	−0.124	−0.024	0.006				
HCE → HP → JC	0.250	0.192	0.318	<0.001				
IS × HCE → OP → JC	−0.001	−0.015	0.016	0.922				
IS × HCE → HP → JC	0.132	0.053	0.174	<0.001				

HCE, Human-AI collaboration engagement; HP, Harmonious passion; IS, Independent self-construal; OP, Obsessive passion; JC, Job crafting; LLCI, Lower-limit of confidence interval; ULCI, Upper-limit of confidence interval, *r*^2^ and *q*^2^ are reported once for each endogenous variable.

To examine the moderating effect, an interaction term (Independent self-construal × human-AI collaboration engagement) was incorporated into the structural model. The results demonstrate that this interaction was significant for harmonious passion (*β* = 0.286, *p* < 0.001). Specifically, under higher levels of independent self-construal, the positive association between human-AI collaboration engagement and harmonious passion becomes stronger, supporting H2a. In contrast, the interaction was not significant for obsessive passion (*β* = 0.005, *p* = 0.917), indicating that H2b was not supported.

To further examine the specific indirect effects implied by the conceptual model, we conducted additional bootstrapping analyses with 5,000 resamples. As shown in Panel C of Table 4, human-AI collaboration engagement had a significant positive indirect effect on job crafting through harmonious passion (*β* = 0.250, *p* < 0.001), whereas it had a significant negative indirect effect on job crafting through obsessive passion (*β* = −0.072, *p* < 0.01). These results indicate that human-AI collaboration engagement is associated with job crafting through two distinct passion-based pathways. In addition, the indirect effect of the interaction term on job crafting through harmonious passion was significant (*β* = 0.132, *p* < 0.001), whereas the indirect effect through obsessive passion was not significant (*β* = −0.001, *p* = 0.922). This pattern is consistent with the earlier moderation results, suggesting that independent self-construal mainly strengthens the harmonious passion pathway rather than the obsessive passion pathway.

### 3.4 Discussion

Study 2 replicates and extends the pattern of associations identified in Study 1 at an overall model level, shifting the analytical focus from motivational experiences to concrete academic work behaviors. The results indicate that the relationships between human-AI collaboration engagement and both harmonious passion and obsessive passion remain consistent across different samples, thereby further strengthening the robustness of the findings from Study 1. Moreover, the differentiation in job crafting associated with distinct types of passion provides important additional insight into the practical implications of human-AI collaboration.

Specifically, harmonious passion is significantly positively associated with job crafting, suggesting that when teachers engage in AI collaboration in a relatively autonomous and flexible manner, they are more likely to proactively reshape their academic practices by adjusting task approaches, workflows, and resource allocation. In contrast, obsessive passion shows a significant negative relationship with job crafting, indicating that even under conditions of high engagement, when AI collaboration is internalized in a controlled and pressure-driven manner, teachers may find it more difficult to actively modify their work methods. This finding suggests that high levels of engagement do not necessarily correspond to greater proactive behavior; rather, behavioral correlates appear to be more closely associated with the quality of motivational experience than with the intensity of engagement per se (Kibaroğlu et al., 2025). By integrating the dualistic model of passion with job crafting behavior, Study 2 shows that human-AI collaboration is associated with markedly different academic practice patterns through distinct forms of passionate experience.

## 4 General discussion

### 4.1 Summary of findings

Drawing on two complementary empirical studies, this research systematically examines university teachers’ psychological experiences in the context of human-AI collaboration and their subsequent behavioral correlates. The findings indicate that engagement in human-AI collaboration does not appear to be associated with academic work experiences in a unidimensional manner. Instead, the observed associations reveal two distinct motivational–behavioral patterns: an empowerment-related pattern characterized by harmonious passion and greater job crafting, and an entrapment-related pattern characterized by obsessive passion and reduced job crafting. At the psychological mechanism level, both studies consistently demonstrate that human-AI collaboration engagement is significantly positively associated with both harmonious passion and obsessive passion. This finding suggests that, during intensive involvement in AI collaboration, university teachers may experience it either as a supportive resource that facilitates academic exploration and self-expression (Renkema and Tursunbayeva, 2024; Zheng et al., 2025) or as an internalized obligation accompanied by psychological pressure and persistent self-imposed demands (Hai et al., 2025; Chuang et al., 2025). This “dual-pathway” pattern corroborates the core proposition of the dualistic model of passion, which posits that the same activity can be internalized in either an autonomous or a controlled manner, thereby producing qualitatively different psychological outcomes (Vallerand et al., 2003; Curran et al., 2015).

Moreover, the findings further illuminate the role of individual differences in the psychological internalization process of human-AI collaboration. Independent self-construal significantly strengthens the positive relationship between human-AI collaboration engagement and harmonious passion. This suggests that when teachers place greater emphasis on autonomy, self-direction, and personal achievement, they are more likely to interpret human-AI collaboration as a resource that supports the expansion of their academic capabilities and the attainment of scholarly goals, a pattern consistent with relatively autonomous motivational experience. Individuals with a more independent self-construal are inclined to integrate work experiences as “self-chosen activities”, thereby fostering positive and harmonious motivational states (Singelis, 1994; Voyer and Franks, 2014). In contrast, independent self-construal does not significantly moderate the relationship between human-AI collaboration engagement and obsessive passion. Interpreted in light of the dualistic model of passion, this finding suggests that the controlled internalization process underlying obsessive passion is less contingent upon whether individuals emphasize autonomy or self-direction, and may instead reflect the pervasive internal pressure and sustained self-imposed demands that commonly emerge in highly engaged collaborative contexts.

Finally, the findings further demonstrate that distinct types of work passion are associated with markedly different patterns of academic practice. Harmonious passion is significantly positively related to job crafting, indicating that when teachers engage in AI collaboration in a relatively autonomous and flexible manner, they are more likely to proactively shape their academic practice by adjusting academic tasks, work processes, and resource allocation. This finding is consistent with prior research conceptualizing job crafting as a key behavioral mechanism through which individuals actively regulate the relationship between their work and themselves (Wrzesniewski and Dutton, 2001; Kibaroğlu et al., 2025). Conversely, obsessive passion is significantly negatively associated with job crafting, suggesting that when AI collaboration is experienced in a more controlled manner, even high levels of engagement may coincide with a reduced tendency to reflect upon and adjust one’s work approaches. This result further underscores that high engagement does not necessarily translate into high proactivity; rather, behavioral outcomes hinge critically on the quality of motivational internalization rather than the intensity of engagement perse.

### 4.2 Theoretical contributions

This study advances theoretical understanding of the relationships among human-AI collaboration, work passion, and academic work behaviors. First, by introducing the dualistic model of passion into the context of university teachers’ human-AI collaboration, this research extends the application of the model to interpret the psychological associations observed in technologically intensive academic work. Prior studies have emphasized that passion reflects the manner in which individuals internalize an activity as part of the self, rather than the intensity of engagement per se (Vallerand et al., 2003; Curran et al., 2015). Building on this perspective, this study shows that engagement in human-AI collaboration is positively associated with both harmonious and obsessive passion, a pattern consistent with autonomous and controlled forms of motivational experience. It addresses limitations in prior research that have largely confined explanations of AI use to outcomes or attitudinal responses, thereby offering a more nuanced account of the motivational processes involved.

Second, this study refines the theoretical understanding of “high engagement” in research on human-AI collaboration. By distinguishing between harmonious passion and obsessive passion, this research shows that under conditions of high involvement in human-AI collaboration, engagement itself does not carry a singular or linear psychological meaning; rather, its psychological correlates may vary depending on how the collaborative experience is interpreted and experienced. Furthermore, the findings reveal that independent self-construal exerts a significant enhancing effect only along the harmonious passion pathway, but does not significantly moderate the obsessive passion pathway. This result suggests that differences in self-construal primarily influence whether human-AI collaboration is autonomously integrated as a “self-chosen academic activity”, thereby amplifying the positive psychological meaning of the collaborative experience. In contrast, within the controlled internalization process associated with obsessive passion, individuals with varying levels of self-construal do not exhibit significant differences. Accordingly, the role of individuals’ self-definitional orientations in the psychological correlates of human-AI collaboration appears to be pathway-specific, with moderating effects concentrated on positive experiences related to autonomous motivational experience rather than on all forms of highly engaged collaborative experiences.

Finally, by linking work passion with job crafting behavior, this study advances theoretical understanding of how psychological experiences are associated with practical responses in academic work. Prior research has noted that university teachers’ academic work is characterized by a high degree of autonomy, with everyday practices often unfolding through continuous job crafting (McNaughtan et al., 2022; Kibaroğlu et al., 2025). The findings of this study indicate that different types of passion play differentiated roles in this process: harmonious passion facilitates proactive adjustments to work approaches, whereas obsessive passion constrains such adaptive behaviors. This finding identifies key psychological antecedents from the perspective of motivational internalization, thereby concretizing the relationship between the dualistic model of passion and academic work practices. By examining the association pattern of “human-AI collaboration engagement—type of passion—job crafting behavior”, this study further elucidates how, in the context of AI’s deep integration into academic work, teachers’ psychological experiences are related to behavioral responses.

### 4.3 Practical implications

For higher education institutions seeking to support faculty members who already use generative AI in academic work, management practices should avoid equating collaboration engagement per se with a positive work state. In contexts that rely heavily on professional judgment and academic identity, human-AI collaboration is often internalized by teachers as a practice closely tied to “how I conduct academic work”. If organizations assume that deeper collaboration is inherently more beneficial, they may overlook the psychological complexity of collaborative experiences and consequently underestimate the potential constraints and pressures embedded within them. Therefore, when promoting AI applications, universities should reconsider the simplistic assumption that greater collaboration is uniformly advantageous, and instead shift their focus from the intensity of collaboration to the quality of the collaborative experience.

On this basis, managerial interventions should not center on further intensifying collaborative involvement, but rather on ensuring that the collaborative process remains adjustable and flexible. Particularly in academic environments that emphasize autonomy and self-direction, teachers are more likely to interpret human-AI collaboration as a resource for achieving personal scholarly goals. However, this does not mean they are naturally insulated from the gradually accumulating internal demands associated with ongoing collaboration. If implicit norms of constant online presence, high-frequency interaction, or immediate responsiveness emerge around AI collaboration, even highly autonomy-oriented teachers may internalize the collaborative experience as a rigid and inescapable self-imposed responsibility.

At a broader level, whether human-AI collaboration can sustainably support the vitality of academic work depends on whether organizations preserve teachers’ discretion to proactively reshape their work practices. When universities formulate AI-related policies for teaching, research, or evaluation, an excessive pursuit of uniform standards or “best practices” may inadvertently standardize collaborative modes and weaken teachers’ capacity to reconstruct the structure, pace, and meaning of their work. In contrast, by institutionally recognizing the legitimacy of diverse academic practice pathways and providing legitimate space for teachers to continuously adjust their modes of human-AI collaboration, organizations can transform the introduction of AI into a sustainable mechanism of academic support rather than a new source of structural pressure.

### 4.4 Limitations and future research

Although this study systematically examined the psychological mechanisms and behavioral consequences of university teachers in the context of human-AI collaboration through two independent samples, several research boundaries remain to be further explored.

First, this study employed a cross-sectional survey design. While this approach is conducive to identifying systematic associations among variables, it is limited in capturing the dynamic evolution of human-AI collaboration, passionate experiences, and work behaviors over time, and it does not allow causal direction or temporal ordering to be established. Future research could adopt longitudinal designs or experience sampling methods to investigate whether teachers’ modes of motivational internalization shift throughout sustained collaboration, as well as to examine the stability and malleability of different types of passion in long-term contexts.

Second, this study focused on Chinese university faculty members who had prior experience using generative AI in academic work, and participants were recruited through purposive, network-based channels. This sampling strategy was appropriate for an initial investigation because the research questions required participants to have direct experience with human-AI collaboration. However, it also limits the generalizability of the findings. The observed associations should therefore not be interpreted as representing academics in general, faculty members who have not used generative AI, or university systems in other national and institutional contexts. Future research could use more diverse sampling strategies and include faculty members with different levels of AI experience across countries, disciplines, and institutional types.

Finally, although this study considered individual-level differences in self-construal, it did not incorporate organizational-level factors such as institutional design or evaluation environments. Future research may adopt multilevel research designs to systematically examine how organizational norms, performance orientations, or modes of technological governance interact with individual motivational internalization processes, thereby offering a more comprehensive understanding of the complex impact of human-AI collaboration on academic and organizational practices.

## 5 Conclusion

As generative AI becomes increasingly embedded in academic work, this study examines university teachers’ psychological experiences and behavioral correlates in human-AI collaboration from the perspective of motivational internalization. By integrating the dualistic model of passion with job crafting behavior, we conceptualize human-AI collaboration as an academic practice that may involve both activation and constraint, rather than as a unidimensional process of technological empowerment. The findings further suggest that the manner in which collaborative experiences are integrated into teachers’ self-concepts may constitute an important psychological correlate of their academic responses. This internalization process is influenced by individuals’ modes of self-definition, yet not all potential constraints can be mitigated by personal traits alone. By examining the association pattern of “human-AI collaboration engagement—type of passion—job crafting behavior”, this study extends prior research that has predominantly centered on tool usage or outcome variables in human-AI collaboration, highlighting the pivotal role of motivational quality—rather than engagement intensity—in understanding academic work practices. Within this specific sample context, the findings offer a clearer perspective on the psychological foundations and practical trajectories of academic work in the age of AI and provide a basis for future research on technological intervention and academic agency.

## Data Availability

The raw data supporting the conclusions of this article will be made available by the authors, without undue reservation.
