# Supplementary material for: When does working with AI empower or entrap university faculty? A dualistic passion perspective on job crafting
Source: Front Psychol. 2026 Aug 28;17:1853560. doi: 10.3389/fpsyg.2026.1853560 (PMC13561786; doi:10.3389/fpsyg.2026.1853560)
Supplement: Supplementary file 1 [file Supplementary_file_1.docx]

Supplementary Material

**Appendix 1.** Common method bias assessment using the common method factor (Study1).

| Construct | Indicator | Substantive Factor Loading(*R1*) | *R1^2^* | Method Factor Loading (*R2*) | *R2^2^* |
| --- | --- | --- | --- | --- | --- |
| Human–AI collaboration engagement(HCE) | HCE1 | 0.717*** | 0.514 | 0.602*** | 0.362 |
|  | HCE2 | 0.732*** | 0.536 | 0.483 | 0.233 |
|  | HCE3 | 0.775*** | 0.601 | -0.242 | 0.059 |
|  | HCE4 | 0.800*** | 0.640 | -0.024 | 0.001 |
|  | HCE5 | 0.772*** | 0.596 | -0.01 | 0.000 |
|  | HCE6 | 0.767*** | 0.588 | -0.148 | 0.022 |
|  | HCE7 | 0.769*** | 0.591 | -0.113 | 0.013 |
|  | HCE8 | 0.778*** | 0.605 | -0.143 | 0.020 |
|  | HCE9 | 0.798*** | 0.637 | -0.226* | 0.051 |
|  | HCE10 | 0.790*** | 0.624 | 0.043*** | 0.002 |
|  | HCE11 | 0.736*** | 0.542 | -0.201* | 0.040 |
| Harmonious passion(HP) | HP1 | 0.852*** | 0.726 | -0.145** | 0.021 |
|  | HP2 | 0.846*** | 0.716 | 0.057 | 0.003 |
|  | HP3 | 0.800*** | 0.640 | 0.106 | 0.011 |
|  | HP4 | 0.828*** | 0.686 | -0.021 | 0.000 |
|  | HP5 | 0.861*** | 0.741 | 0.005 | 0.000 |
|  | HP6 | 0.822*** | 0.676 | -0.013 | 0.000 |
|  | HP7 | 0.848*** | 0.719 | 0.016 | 0.000 |
| Independent self-construal(IS) | IS1 | 0.879*** | 0.773 | -0.021 | 0.000 |
|  | IS2 | 0.904*** | 0.817 | 0.018 | 0.000 |
|  | IS3 | 0.80*** | 0.640 | -0.022 | 0.000 |
|  | IS4 | 0.812*** | 0.659 | 0.010 | 0.000 |
|  | IS5 | 0.851*** | 0.724 | 0.012 | 0.000 |
|  | IS6 | 0.819*** | 0.671 | 0.102* | 0.010 |
|  | IS7 | 0.822*** | 0.676 | 0.051 | 0.003 |
|  | IS8 | 0.825*** | 0.681 | -0.011 | 0.000 |
|  | IS9 | 0.819*** | 0.671 | -0.002 | 0.000 |
|  | IS10 | 0.822*** | 0.676 | -0.073 | 0.005 |
|  | IS11 | 0.804*** | 0.646 | -0.056 | 0.003 |
|  | IS12 | 0.858*** | 0.736 | -0.010 | 0.000 |
| Obsessive passion(OP) | OP1 | 0.832*** | 0.692 | -0.047 | 0.002 |
|  | OP2 | 0.820*** | 0.672 | -0.007 | 0.000 |
|  | OP3 | 0.835*** | 0.697 | 0.017 | 0.000 |
|  | OP4 | 0.789*** | 0.623 | -0.07 | 0.005 |
|  | OP5 | 0.869*** | 0.755 | 0.021 | 0.000 |
|  | OP6 | 0.805*** | 0.648 | 0.032 | 0.001 |
|  | OP7 | 0.821*** | 0.674 | 0.051 | 0.003 |
| Average | | 0.813 | 0.662 | 0.001 | 0.024 |
| *** p < 0.001, ** p < 0.01, * p < 0.05 | | | | | |

**Appendix 2.** Common method bias assessment using the common method factor (Study2).

| Construct | Indicator | Substantive Factor Loading(*R1*) | *R1^2^* | Method Factor Loading (*R2*) | *R2^2^* |
| --- | --- | --- | --- | --- | --- |
| Human–AI collaboration engagement(HCE) | HCE1 | 0.722*** | 0.521 | 0.403* | 0.162 |
|  | HCE2 | 0.742*** | 0.551 | 0.426*** | 0.181 |
|  | HCE3 | 0.715*** | 0.511 | 0.293** | 0.086 |
|  | HCE4 | 0.775*** | 0.601 | -0.059 | 0.003 |
|  | HCE5 | 0.771*** | 0.594 | -0.066 | 0.004 |
|  | HCE6 | 0.755*** | 0.570 | -0.215** | 0.046 |
|  | HCE7 | 0.775*** | 0.601 | -0.115 | 0.013 |
|  | HCE8 | 0.795*** | 0.632 | -0.109 | 0.012 |
|  | HCE9 | 0.800*** | 0.640 | -0.286** | 0.082 |
|  | HCE10 | 0.806*** | 0.650 | -0.078 | 0.006 |
|  | HCE11 | 0.725*** | 0.526 | -0.171 | 0.029 |
| Harmonious passion(HP) | HP1 | 0.824*** | 0.679 | -0.151** | 0.023 |
|  | HP2 | 0.850*** | 0.723 | 0.033 | 0.001 |
|  | HP3 | 0.831*** | 0.691 | 0.08 | 0.006 |
|  | HP4 | 0.812*** | 0.659 | 0.031 | 0.001 |
|  | HP5 | 0.841*** | 0.707 | 0.018 | 0.000 |
|  | HP6 | 0.835*** | 0.697 | 0.049 | 0.002 |
|  | HP7 | 0.825*** | 0.681 | -0.063 | 0.004 |
| Independent self-construal(IS) | IS1 | 0.897*** | 0.805 | -0.015 | 0.000 |
|  | IS2 | 0.845*** | 0.714 | -0.007 | 0.000 |
|  | IS3 | 0.851*** | 0.724 | -0.01 | 0.000 |
|  | IS4 | 0.849*** | 0.721 | -0.05 | 0.003 |
|  | IS5 | 0.904*** | 0.817 | 0.022 | 0.000 |
|  | IS6 | 0.853*** | 0.728 | 0.013 | 0.000 |
|  | IS7 | 0.849*** | 0.721 | 0.041 | 0.002 |
|  | IS8 | 0.827*** | 0.684 | 0.003 | 0.000 |
|  | IS9 | 0.821*** | 0.674 | -0.037 | 0.001 |
|  | IS10 | 0.834*** | 0.696 | 0.022 | 0.000 |
|  | IS11 | 0.813*** | 0.661 | 0.028 | 0.001 |
|  | IS12 | 0.862*** | 0.743 | -0.011 | 0.000 |
| Job crafting(JC) | JC1 | 0.771*** | 0.594 | 0.089* | 0.008 |
|  | JC2 | 0.790*** | 0.624 | 0.062 | 0.004 |
|  | JC3 | 0.895*** | 0.801 | -0.045 | 0.002 |
|  | JC4 | 0.851*** | 0.724 | -0.053 | 0.003 |
|  | JC5 | 0.861*** | 0.741 | -0.037 | 0.001 |
|  | JC6 | 0.732*** | 0.536 | -0.003 | 0.000 |
| Obsessive passion(OP) | OP1 | 0.816*** | 0.666 | -0.049 | 0.002 |
|  | OP2 | 0.835*** | 0.697 | -0.041 | 0.002 |
|  | OP3 | 0.827*** | 0.684 | -0.032 | 0.001 |
|  | OP4 | 0.809*** | 0.654 | 0.077 | 0.006 |
|  | OP5 | 0.846*** | 0.716 | 0.005 | 0.000 |
|  | OP6 | 0.811*** | 0.658 | -0.011 | 0.000 |
|  | OP7 | 0.795*** | 0.632 | 0.054 | 0.003 |
| Average | | 0.815 | 0.666 | 0.001 | 0.016 |
| *** p < 0.001, ** p < 0.01, * p < 0.05 | | | | | |

**Appendix 3.** Analysis results of the marker variable method (Study1).

| Path | *β*-value | *t*-value | *p*-value | *R^2^* | Adjusted *R^2^* |
| --- | --- | --- | --- | --- | --- |
| The result before adding the marker variable. | | | | | |
| HCE → HP | 0.544 | 11.185 | < 0.001 |  |  |
| HCE → OP | 0.465 | 8.280 | < 0.001 |  |  |
| IS x HCE →HP | 0.275 | 4.018 | < 0.001 | 0.389 | 0.374 |
| IS x HCE → OP | 0.017 | 0.281 | 0.779 | 0.227 | 0.208 |
| The result after adding the marker variable. | | | | | |
| HCE →HP | 0.548 | 11.108 | < 0.001 |  |  |
| HCE →OP | 0.463 | 8.173 | < 0.001 |  |  |
| IS x HCE→ HP | 0.290 | 4.134 | < 0.001 | 0.397 | 0.380 |
| IS x HCE → OP | 0.009 | 0.16 | 0.873 | 0.230 | 0.207 |
| MV → HP | 0.094 | 1.377 | 0.169 |  |  |
| MV→ OP | -0.047 | 0.666 | 0.506 |  |  |
| *HCE* Human–AI collaboration engagement, *HP* Harmonious passion, *IS* Independent self-construal, *OP* Obsessive passion, *JC* Job crafting, *MV* Marker variable. | | | | | |

**Appendix 4.** Analysis results of the marker variable method (Study2).

| Path | *β*-value | *t*-value | *p*-value | *R^2^* | Adjusted *R^2^* |
| --- | --- | --- | --- | --- | --- |
| The result before adding the marker variable. | | | | | |
| HCE →HP | 0.540 | 12.959 | < 0.001 |  |  |
| HCE →OP | 0.517 | 12.023 | < 0.001 |  |  |
| HP →JC | 0.462 | 10.312 | < 0.001 |  |  |
| OP →JC | -0.139 | 2.905 | 0.004 | 0.191 | 0.179 |
| IS x HCE →HP | 0.286 | 4.859 | < 0.001 | 0.393 | 0.388 |
| IS x HCE → OP | 0.005 | 0.104 | 0.917 | 0.269 | 0.262 |
| The result after adding the marker variable. | | | | | |
| HCE → HP | 0.540 | 12.959 | < 0.001 |  |  |
| HCE→ OP | 0.512 | 11.791 | < 0.001 |  |  |
| HP → JC | 0.462 | 10.268 | < 0.001 |  |  |
| OP→ JC | -0.136 | 2.83 | 0.005 | 0.192 | 0.178 |
| IS x HCE → HP | 0.287 | 4.825 | < 0.001 | 0.393 | 0.386 |
| IS x HCE → OP | 0.011 | 0.227 | 0.821 | 0.271 | 0.263 |
| MV→ HP | 0.008 | 0.137 | 0.891 |  |  |
| MV → JC | -0.031 | 0.414 | 0.679 |  |  |
| MV → OP | 0.053 | 1.010 | 0.313 |  |  |
| *HCE* Human–AI collaboration engagement, *HP* Harmonious passion, *IS* Independent self-construal, *OP* Obsessive passion, *JC* Job crafting, *MV* Marker variable. | | | | | |

**Appendix 5.** Model fit results

| Indicators | Study1 | | Study2 | |
| --- | --- | --- | --- | --- |
|  | Saturated model | Estimated model | Saturated model | Estimated model |
| SRMR | 0.053 | 0.054 | 0.046 | 0.049 |
| d_ULS | 2.323 | 2.381 | 2.308 | 2.603 |
| d_G | 1.283 | 1.287 | 1.081 | 1.092 |
| Chi-square | 1631.618 | 1639.297 | 2098.888 | 2127.398 |
| NFI | 0.798 | 0.797 | 0.838 | 0.836 |

**Appendix 6.** Results of *f² e*ffect sizes (Study1).

| Paths | *f^2^* |
| --- | --- |
| AI usage frequency → HP | 0.011 |
| AI usage frequency → OP | 0.004 |
| Age →HP | 0.000 |
| Age → OP | 0.002 |
| Gender → HP | 0.000 |
| Gender → OP | 0.006 |
| HCE →HP | 0.482 |
| HCE→ OP | 0.279 |
| IS → HP | 0.014 |
| IS → OP | 0.000 |
| IS x HCE → HP | 0.125 |
| IS x HCE → OP | 0.000 |
| *HCE* Human–AI collaboration engagement, *HP* Harmonious passion, *IS* Independent self-construal, *OP* Obsessive passion. | |

**Appendix 7.** Results of *f² e*ffect sizes (Study2).

| Paths | *f^2^* |
| --- | --- |
| AI usage frequency → JC | 0.001 |
| Age → JC | 0.001 |
| Gender → JC | 0.001 |
| HCE →HP | 0.476 |
| HCE → OP | 0.361 |
| HP → JC | 0.231 |
| IS → HP | 0.007 |
| IS → OP | 0.000 |
| OP → JC | 0.021 |
| IS x HCE → HP | 0.122 |
| IS x HCE → OP | 0.000 |
| *HCE* Human–AI collaboration engagement, *HP* Harmonious passion, *IS* Independent self-construal, *OP* Obsessive passion, *JC* Job crafting. | |

**Appendix 8.** Analysis results of control variables (Study1).

| Hypotheses path | *β*-values | *t*-values | LLCI | ULCI | *p*-value |
| --- | --- | --- | --- | --- | --- |
| AI usage frequency → HP | -0.083 | 1.634 | -0.185 | 0.016 | 0.102 |
| AI usage frequency→ OP | 0.057 | 1.088 | -0.048 | 0.161 | 0.277 |
| Age → HP | 0.004 | 0.078 | -0.093 | 0.108 | 0.938 |
| Age → OP | 0.045 | 0.832 | -0.059 | 0.149 | 0.405 |
| Gender → HP | 0.002 | 0.016 | -0.207 | 0.198 | 0.987 |
| Gender → OP | -0.135 | 1.179 | -0.362 | 0.091 | 0.238 |
| *HP* Harmonious passion, *IS* Independent self-construal, *OP* Obsessive passion, *LLCI* Lower-limit of confidence interval, *ULCI* Upper-limit of confidence interval. | | | | | |

**Appendix 9.** Analysis results of control variables (Study2).

| Hypotheses path | *β*-values | *t*-values | LLCI | ULCI | *p*-value |
| --- | --- | --- | --- | --- | --- |
| AI usage frequency → JC | -0.032 | 0.673 | -0.122 | 0.061 | 0.501 |
| Age → JC | -0.028 | 0.549 | -0.128 | 0.075 | 0.583 |
| Gender → JC | -0.049 | 0.501 | -0.246 | 0.14 | 0.616 |
| *JC* Job crafting, *LLCI* Lower-limit of confidence interval, *ULCI* Upper-limit of confidence interval. | | | | | |

**Appendix 10.** PLSpredict results (Study1).

| Construct | Indicator | *Q²*predict | PLS-SEM RMSE | LM RMSE | RMSE Difference | PLS-SEM MAE | LM MAE | MAE Difference | Result |
| --- | --- | --- | --- | --- | --- | --- | --- | --- | --- |
| HP | HP1 | 0.179 | 1.577 | 1.701 | -0.124 | 1.274 | 1.362 | -0.088 | Acceptable |
| HP | HP2 | 0.270 | 1.436 | 1.537 | -0.101 | 1.174 | 1.245 | -0.071 | Acceptable |
| HP | HP3 | 0.264 | 1.542 | 1.614 | -0.072 | 1.272 | 1.300 | -0.028 | Acceptable |
| HP | HP4 | 0.219 | 1.640 | 1.772 | -0.132 | 1.353 | 1.442 | -0.089 | Acceptable |
| HP | HP5 | 0.245 | 1.510 | 1.637 | -0.127 | 1.216 | 1.312 | -0.096 | Acceptable |
| HP | HP6 | 0.237 | 1.275 | 1.355 | -0.080 | 0.999 | 1.051 | -0.052 | Acceptable |
| HP | HP7 | 0.267 | 1.553 | 1.688 | -0.135 | 1.288 | 1.392 | -0.104 | Acceptable |
| OP | OP1 | 0.118 | 1.679 | 1.714 | -0.035 | 1.370 | 1.398 | -0.028 | Acceptable |
| OP | OP2 | 0.145 | 1.648 | 1.684 | -0.036 | 1.345 | 1.371 | -0.026 | Acceptable |
| OP | OP3 | 0.100 | 1.691 | 1.684 | 0.007 | 1.335 | 1.333 | 0.002 | Weak |
| OP | OP4 | 0.069 | 1.426 | 1.484 | -0.058 | 1.131 | 1.175 | -0.044 | Acceptable |
| OP | OP5 | 0.127 | 1.637 | 1.639 | -0.002 | 1.322 | 1.302 | 0.020 | Partially acceptable |
| OP | OP6 | 0.123 | 1.544 | 1.605 | -0.061 | 1.243 | 1.299 | -0.056 | Acceptable |
| OP | OP7 | 0.143 | 1.705 | 1.761 | -0.056 | 1.413 | 1.451 | -0.038 | Acceptable |
| All *Q²*predict values were above zero, indicating predictive relevance. Most RMSE and MAE differences were negative, suggesting that the PLS-SEM model generally outperformed the LM benchmark. However, OP3 showed slightly higher RMSE and MAE values than the LM benchmark, while OP5 showed a slightly higher MAE value. Overall, the model demonstrated acceptable predictive power. | | | | | | | | | |

**Appendix 11.** PLSpredict results (Study2).

| Construct | Indicator | *Q²*predict | PLS-SEM RMSE | LM RMSE | RMSE Difference | PLS-SEM MAE | LM MAE | MAE Difference | Result |
| --- | --- | --- | --- | --- | --- | --- | --- | --- | --- |
| HP | HP1 | 0.194 | 1.564 | 1.666 | -0.102 | 1.262 | 1.336 | -0.074 | Acceptable |
| HP | HP2 | 0.274 | 1.473 | 1.562 | -0.089 | 1.192 | 1.253 | -0.061 | Acceptable |
| HP | HP3 | 0.3 | 1.476 | 1.551 | -0.075 | 1.214 | 1.275 | -0.061 | Acceptable |
| HP | HP4 | 0.241 | 1.299 | 1.353 | -0.054 | 1.028 | 1.081 | -0.053 | Acceptable |
| HP | HP5 | 0.275 | 1.55 | 1.64 | -0.09 | 1.264 | 1.331 | -0.067 | Acceptable |
| HP | HP6 | 0.279 | 1.493 | 1.61 | -0.117 | 1.241 | 1.311 | -0.07 | Acceptable |
| HP | HP7 | 0.246 | 1.544 | 1.657 | -0.113 | 1.282 | 1.342 | -0.06 | Acceptable |
| JC | JC1 | 0.065 | 1.659 | 1.74 | -0.081 | 1.366 | 1.424 | -0.058 | Acceptable |
| JC | JC2 | 0.054 | 1.692 | 1.756 | -0.064 | 1.407 | 1.442 | -0.035 | Acceptable |
| JC | JC3 | 0.045 | 1.776 | 1.835 | -0.059 | 1.466 | 1.514 | -0.048 | Acceptable |
| JC | JC4 | 0.021 | 1.535 | 1.586 | -0.051 | 1.289 | 1.314 | -0.025 | Acceptable |
| JC | JC5 | 0.033 | 1.528 | 1.599 | -0.071 | 1.256 | 1.314 | -0.058 | Acceptable |
| JC | JC6 | 0.026 | 1.405 | 1.463 | -0.058 | 1.127 | 1.172 | -0.045 | Acceptable |
| OP | OP1 | 0.149 | 1.584 | 1.614 | -0.03 | 1.291 | 1.315 | -0.024 | Acceptable |
| OP | OP2 | 0.163 | 1.532 | 1.542 | -0.01 | 1.244 | 1.254 | -0.01 | Acceptable |
| OP | OP3 | 0.127 | 1.515 | 1.531 | -0.016 | 1.181 | 1.212 | -0.031 | Acceptable |
| OP | OP4 | 0.19 | 1.511 | 1.571 | -0.06 | 1.172 | 1.225 | -0.053 | Acceptable |
| OP | OP5 | 0.181 | 1.495 | 1.529 | -0.034 | 1.223 | 1.248 | -0.025 | Acceptable |
| OP | OP6 | 0.166 | 1.516 | 1.539 | -0.023 | 1.22 | 1.231 | -0.011 | Acceptable |
| OP | OP7 | 0.191 | 1.561 | 1.61 | -0.049 | 1.275 | 1.294 | -0.019 | Acceptable |
| All *Q²*predict values were above zero. In addition, all RMSE and MAE differences were negative, indicating that the PLS-SEM model produced lower prediction errors than the LM benchmark for all endogenous indicators. Therefore, the model demonstrated acceptable out-of-sample predictive power. | | | | | | | | | |

**Appendix 12.** The measurement items of this study.

| Construct | Item |
| --- | --- |
| Human–AI collaboration engagement  (HCE) | 1.I respect the opinions provided by AI tools when I use them for my academic work. |
|  | 2.If the AI does not understand my request, I rephrase my question. |
|  | 3.I consult AI when I need additional information for teaching or research. |
|  | 4.If I have difficulty understanding a topic, I ask AI for help. |
|  | 5.I use AI for brainstorming ideas related to my academic work. |
|  | 6.I break down complex questions into simpler ones so that AI can better understand me. |
|  | 7.When I need to learn something new for my work, I use AI. |
|  | 8.I use AI to help manage my academic tasks or time. |
|  | 9.I am willing to pay to use AI tools. |
|  | 10.I adjust my work processes to better integrate AI into my academic activities. |
|  | 11.I consult AI before making important academic or work-related decisions. |
| Job crafting(JC) | 1.I introduce new approaches on my own to improve my teaching or academic work. |
|  | 2.I independently change minor work procedures that I find inefficient. |
|  | 3.On my own, I change the way I perform my academic tasks to make my work easier. |
|  | 4.I independently reorganize teaching or work-related resources to better support my work. |
|  | 5.I independently organize new academic or instructional activities related to my work. |
|  | 6.On my own, I introduce external tools or resources (e.g., digital tools or platforms) to support my teaching or research. |
| Harmonious passion(HP) | 1.Using AI in my academic work allows me to experience a variety of new possibilities. |
|  | 2.The new things I discover through using AI make me appreciate it even more. |
|  | 3.Using AI allows me to have memorable and positive work experiences. |
|  | 4.Using AI reflects qualities that I value about myself as a scholar or teacher. |
|  | 5.Using AI is in harmony with my other academic and personal activities. |
|  | 6.For me, using AI is a passion that I am still able to control. |
|  | 7.I feel deeply engaged when I use AI for my academic work. |
| Obsessive passion(OP) | 1.I feel that I cannot carry out my academic work without using AI. |
|  | 2.The urge to use AI is so strong that I find it hard to resist. |
|  | 3.I have difficulty imagining my academic work without AI. |
|  | 4.I feel emotionally dependent on using AI for my work. |
|  | 5.I have a hard time controlling my need to use AI. |
|  | 6.I sometimes feel almost obsessed with using AI. |
|  | 7.My mood depends on whether I am able to use AI in my work. |
| Independent self-construal(IS) | 1.I’d rather say “No” directly than risk being misunderstood. |
|  | 2.Speaking up during a class is not a problem for me. |
|  | 3.Having a lively imagination is important to me. |
|  | 4.I am comfortable with being singled out for praise or rewards. |
|  | 5.I am the same person at home that I am at school. |
|  | 6.Being able to take care of myself is a primary concern for me. |
|  | 7.I act the same way no matter who I am with. |
|  | 8.I feel comfortable using someone’s first name soon after I meet them, even when they are much older than I am. |
|  | 9.I prefer to be direct and forthright when dealing with people I’ve just met. |
|  | 10.I enjoy being unique and different from others in many respects. |
|  | 11.My personal identity, independent of others, is very important to me. |
|  | 12.I value being in good health above everything. |
| Marker variable: general life preference orientation(MV) | 1.I prefer having a regular daily routine rather than frequent changes in my everyday life. |
|  | 2.I enjoy planning ordinary daily activities in advance. |
|  | 3.I feel more comfortable when my daily life follows a stable pattern. |
|  | 4.I value consistency in my everyday habits. |
|  | 5.I like to keep my daily lifestyle predictable. |
